# Intelligent Dynamic Quality Prediction of Chilled Chicken with Integrated IoT Flexible Sensing and Knowledge Rules Extraction

**DOI:** 10.3390/foods11060836

**Published:** 2022-03-15

**Authors:** Jinchao Xu, Ruiqin Ma, Stevan Stankovski, Xue Liu, Xiaoshuan Zhang

**Affiliations:** 1 Beijing Laboratory of Food Quality and Safety, College of Engineering, China Agricultural University, Beijing 100083, China; xuvic@cau.edu.cn; 2 National Research Faculty for Phenotypic and Genotypic Analysis of Model Animals, China Agricultural University, Beijing 100083, China; maruiqin@cau.edu.cn; 3 Faculty of Technical Sciences, University of Novi Sad, 21000 Novi Sad, Serbia; stevan@uns.ac.rs; 4 Beijing Laboratory of Food Quality and Safety, College of Information and Electrical Engineering, China Agricultural University, Beijing 100083, China; liusnow@cau.edu.cn

**Keywords:** chilled chicken, intelligent dynamic prediction model, flexible sensing, knowledge rules, quality evaluation standard

## Abstract

With the enhancement of consumers’ food safety awareness, consumers have become more stringent on meat quality. This study constructs an intelligent dynamic prediction model based on knowledge rules and integrates flexible humidity sensors into the non-destructive monitoring of the Internet of Things to provide real-time feedback and dynamic adjustments for the chilled chicken cold chain. The optimized sensing equipment can be attached to the inside of the packaging to deal with various abnormal situations during the cold chain, effectively improving the packaging effect. Through correlation analysis of collected data and knowledge rule extraction of critical factors in the cold chain, the established quality evaluation and prediction model achieved detailed chilled chicken quality level classification and intelligent quality prediction. The obtained results show that the accuracy of the prediction model is higher than 90.5%, and all the regression coefficients are close to 1.00. The relevant personnel (workers and cold chain managers) were invited to participate in the performance analysis and optimization suggestion to improve the applicability of the established prediction model. The optimized model can provide a more efficient theoretical reference for timely decision-making and further e-commerce management.

## 1. Introduction

Nowadays, with the improvement of living standards, consumers’ demand for “fresher” food poses major challenges for food quality and safety [1,2]. Chilled meat refers to that which is always maintained at a low temperature of 0–4 °C during cold chain storage, transportation, and sales to ensure its quality and freshness [3]. Generally, temperature fluctuation is the main reason for the deterioration of chilled meat due to microbial contamination [4,5]. In addition, humidity, oxygen [6], carbon dioxide [7], and ammonia [8] are also closely related to the chilled meat quality. Currently, electronic noses [9], TTI [10] and intrusive sensors [11] are mostly used to indicate the relationship with meat quality. There is great interest among members of the food supply chain in developing cost-effective, reliable, and intelligent methods to evaluate the chilled meat quality [12].

Dynamic non-destructive monitoring based on artificial intelligence and the Internet of Things (IoT) is a new technological development [13] that is gradually replacing cumbersome and intrusive measurement of traditional handheld instruments. It has been widely applied to packaged food in the cold chain process due to its advantages in real-time, continuous, and dynamic monitoring [14,15]. The flexible sensors that operate at packaging have attracted more attention due to pliability and low power consumption [16]. Moreover, the techniques used in the flexible gas sensing industry, such as inkjet printing, enable the large-scale fabrication of low-cost effective systems [17]. By integrating flexible sensors, battery-less power supply, and wireless transmission [18,19], etc., it can effectively deal with various complex and special conditions of packaged food during the cold chain such as packaging deformation and size optimization.

Additionally, as a mathematical method with strong self-learning, self-adaptation, and other abilities [20], the BP neural network has been widely used in various food supply chains [21,22]. Whereas, conventional quality evaluation and the BP neural network have not realized the comprehensive evaluation and prediction of the critical influencing factors in the cold chain, resulting in indistinct and oversimplified final results [23]. As a consequence, the introduction of intelligent evaluation methods into the Internet of Things is considered to improve the accuracy of final results. Due to the uniqueness and diversity of the cold chain process, knowledge rules provide a comprehensive analysis to ensure the correct execution of the processes [24]. It is adopted as a cost-effective way to provide accurate evaluation and system optimization, which stores a set of rules for management support, expert knowledge, and so on [25]. By determining the strong relationship between the data collected during the process execution and the knowledge rules [26], an optimized quality evaluation standard and prediction model is constructed [27] to effectively ensure the quality and safety of products in the actual process and provide decision-makers and users with a more accurate evaluation and prediction results.

In China’s national standards, Volatile base nitrogen and sensory evaluation are regarded as important factors indicating chilled chicken quality and shelf life. Nevertheless, according to GB 2707-2016 “Chicken Hygienic Standards” with other countries’ evaluation standards for chicken quality, the current evaluation under different conditions is vague and oversimplified, which requires more accurate evaluation standards and prediction methods to avoid invasive tests, especially in a confined space such as cold chain transportation.

Based on previous studies, this study proposes an intelligent dynamic quality prediction of chilled chicken with integrated IoT flexible sensing and knowledge rules extraction in the cold chain. The monitoring equipment collects critical parameters through integrated optimized sensors and the relevant processed data is analyzed statistically to evaluate chilled chicken quality in level. Additionally, this paper used conventional quality evaluation methods as the basic knowledge framework, considering the impact of other rules on the quality of chilled chicken [28], and constructed an intelligent dynamic quality prediction model based on knowledge rules and evaluation standards. The Discussion and Conclusion about the implementation of model performance are provided at the end to verify evaluation standard and prediction effect. This research has made the following contributions to the research field of cold chain logistics:

(1) Integrate flexible sensing into IoT to optimize packaging effects and deal with complex cold chain monitoring;
(2) Construct an intelligent chilled chicken quality prediction model based on knowledge rules framework and quality evaluation standard through process analysis, collected relevant data analysis, and quality indicators analysis.

## 2. Materials and Methods

### 2.1. Conceptual Framework for Intelligent Dynamic Quality Prediction Model

**Figure 1:**
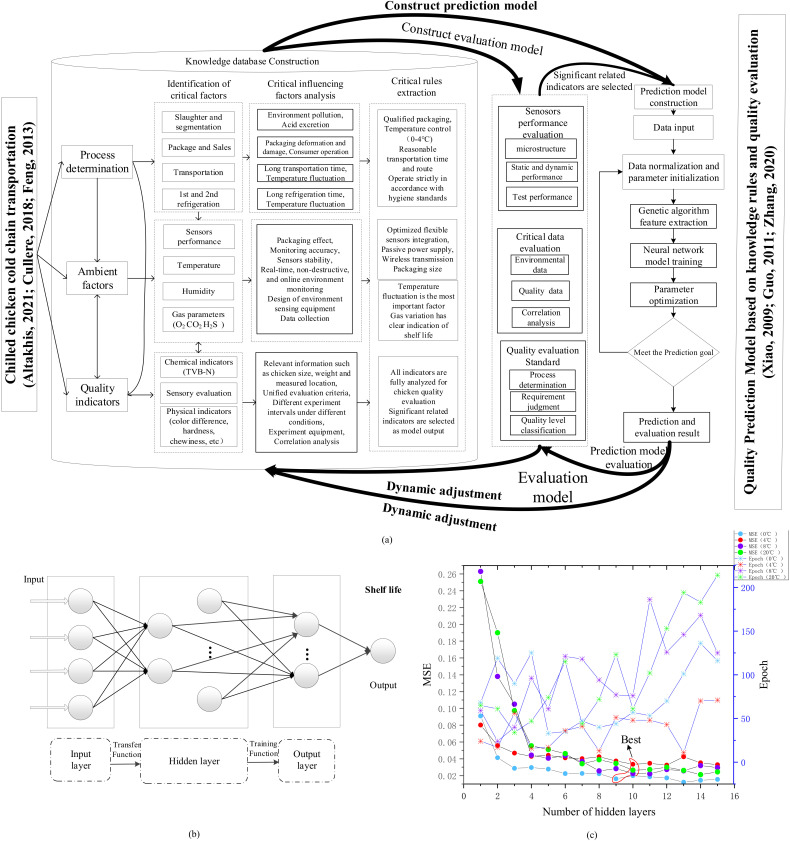
Intelligent dynamic quality prediction model based on knowledge rules and evaluation standard. (**a**) Model construction; (**b**) Prediction model design; (**c**) Prediction model parameter optimization.

The intelligent dynamic quality prediction model based on knowledge rules and evaluation criteria is shown in Figure 1a. The normal implementation of chilled chicken cold chain transportation is influenced by various factors. Through preliminary investigation and reference [4,29,30], various critical factors (such as sensor performance, gas change and physical and chemical indicators, etc.) were classified and identified as: process determination, ambient factors, and quality indicators. Figure 1a summarized all the evaluated indicators (such as temperature change, cold chain transportation, and sensory evaluation, etc.) in this experiment. As the knowledge base considered the impact of hazard analysis of each node in the cold chain, micro-environmental factors and quality indicators on final results, the knowledge rules affecting the normal operation of the cold chain, such as process analysis, packaging requirements, sensor design, parameter monitoring, and preparation and implementation of quality experiments, etc., were extracted, respectively, to construct and supplement the final knowledge rules database. According to the knowledge rules extracted from the database, temperature fluctuation is the most important factor during the cold chain process and should be strictly controlled. Additionally, the optimized flexible sensor integration can effectively reduce the packaging size and improve the packaging effect while maintaining monitoring accuracy. Finally, the quality indicators and environmental signals with high correlation are selected for subsequent quality evaluation and prediction modeling.

Based on the extracted knowledge rules, sensor performance evaluation integrated with flexible sensing, statistical analysis and evaluation of collected critical data, and selection of significantly related indicators to establish evaluation standard together constituted the evaluation model and provided knowledge rules for subsequent quality prediction. Simultaneously, the results of the prediction were re-output to the evaluation model to evaluate the effect of the prediction model and transmit all the evaluation results to the knowledge rule database to improve the accuracy of the next evaluation and prediction.

According to the knowledge rule base and constructed evaluation standard, the constructed prediction model could intelligently and dynamically predict the quality of chilled chicken according to the critical parameters collected in real-time to obtain accurate quality results. The BP neural network is a multilayer feedforward neural network [31], which can solve the nonlinear relationship between different indicators [32]. The conceptual structure of the prediction model was generally composed of an input layer, a hidden layer, and an output layer (Figure 1b). At first, the input signal was processed layer by layer during the signal propagation process. If the expected output error was too large, it would turn to back propagation, adjust the weight and threshold according to the prediction error [33]. The optimization of neural network parameters is shown in Figure 1c. In this study, the commonly used genetic algorithm was selected to optimize the BP neural network and improve its adaptability. The MATLAB R2018b math software (version 9.1, MathWorks Inc., Natick, MA, USA) was used for modeling. The settings and training of related parameters were as follows: the momentum factor was set to the default 0.9, the learning rate was set to 0.05, and the training accuracy was set to 0.000001. Through continuous optimization of parameters under different conditions, the neural network model had relatively higher prediction performance (minimum mean square error) and a more ideal fitting effect (fewer iterations) at different temperatures. After many times of training, the predicted and expected output will continue to approach.

### 2.2. Non-Destructive Monitoring Equipment Integrated with Flexible Sensor

**Figure 2:**
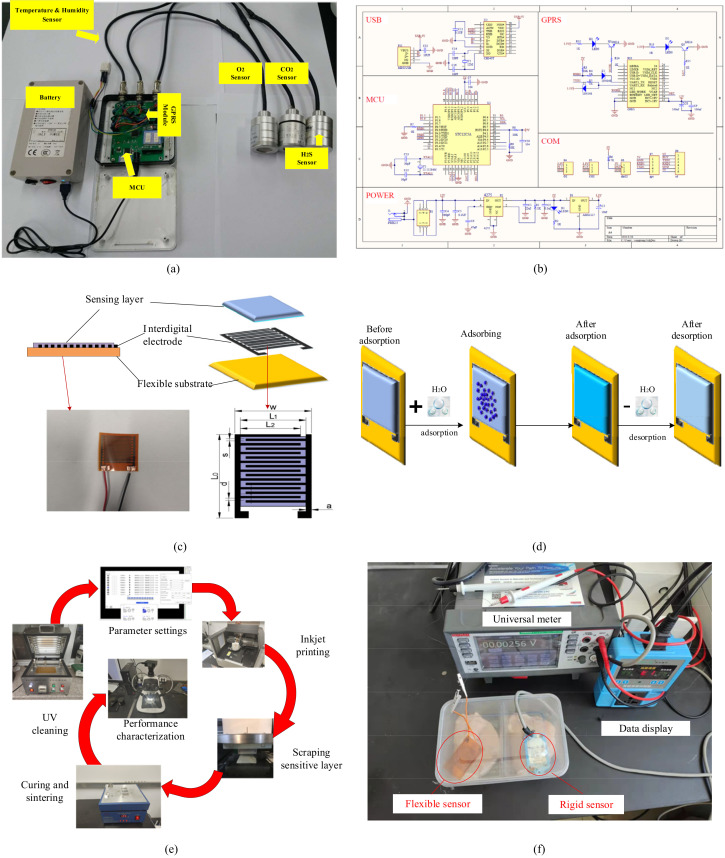
The monitoring equipment architecture. (**a**) The hardware architecture; (**b**) Multi-parameter information collection circuit diagram; (**c**) Flexible humidity sensor block diagram; (**d**) Flexible humidity sensing mechanism; (**e**) Flexible sensor fabrication process; (**f**) Comparison experiment between flexible humidity sensor and conventional sensor.

Conventional monitoring equipment is likely to lead to contamination and affect monitoring accuracy because of unreasonable sensor layout and large packaging size. The designed hardware architecture consists of four modules that include a power supply module, a sensing module, an information processing module, and a wireless communication module (Figure 2a). The relevant multi-parameter information collection circuit diagram is shown in Figure 2b.

The sensing module captured the environmental information and geographic parameters during the cold chain by using a temperature and humidity sensor, gas sensor, and GPS device. The parameter performance of relevant sensors is listed in Table 1. The processing module processed the data received from the sensing module at regular intervals. After filtering and A/D conversion, the processed data was sent to the data analysis and application module via GPRS wireless communication module. The data analysis and application module were responsible for performing analysis and interactive functions which consists of a server module and an application module. It not only provided real-time information warnings and feedback treatment measures but also provided administrators with historical data and simplified operation and configuration interfaces. The power module continued to supply power to the device during the entire working process.

Meanwhile, this paper also proposes a flexible humidity sensor (Figure 2c) to improve architecture performance. The flexible thin-film resistive sensor is composed of a sensing layer, interdigital electrode, and flexible substrate. As shown in Figure 2d, the sensing layer sensed the humidity signal directly and converted it into an electrical signal that had a definite relationship with the measured. The sensing layer underwent an adsorption reaction with water molecules in the air and the electrical conductivity changes in a negative correlation with the environmental humidity. Then the interdigital electrode converted the conductivity of the sensing layer into an output current signal. Finally, the environmental humidity was reflected by the change of resistance value. In this experiment, we used Polyethylene terephthalate (PET) flexible film as the substrate for inkjet printing deposition of the electrode and sensitive materials. The specific preparation process of the flexible sensor is shown in Figure 2e, including substrate ultraviolet cleaning, printing parameter setting, interdigital electrode inkjet printing, dispensing and scraping sensitive layer, curing and sintering after fabrication and performance characterization. All materials were provided by Shanghai Mifang Electronic Technology Co., Ltd., Shanghai, China. A control experiment of the flexible humidity sensor and rigid sensor was proposed to evaluate the improved sensor performance (Figure 2f).

### 2.3. Experiment Design and Scenario Implementation

According to the field survey of the actual cold chain logistics, an experimental simulation of the entire chilled chicken cold chain process was conducted in the laboratory. Three hundred pieces (150 g/piece) of chilled chicken breasts were randomly divided and put into the numbered (1–4) test box (40 cm × 30 cm × 50 cm), which was sealed with polyethylene heat shrinkable film. For packaged chilled chicken at 0 °C and 4 °C, the quality indicators were measured every 24 h. As for chilled chicken at 8 °C and 20 °C, they were measured every 12 h and every 8 h, respectively. Finally, the actual cold chain and variable temperature experiment (packaging and sales at 20 °C, 1st refrigeration at 8 °C, 2nd refrigeration and transfer at 4 °C, and transportation at 0 °C) were implemented to verify the quality evaluation standard and intelligent quality prediction effects. The specific parameters were determined as follows.

#### 2.3.1. Critical Environmental Parameters

In this experiment, temperature and humidity, oxygen, carbon dioxide and hydrogen sulfide were used to sense environmental parameters. The sensors were placed in the top of the box during the packaging stage to avoid contact with the chilled chicken. At the same time, they collected and monitored the temperature, humidity and gas environment changes in the headspace of the package every 10 min dynamically.

#### 2.3.2. Color Difference

During the entire cold chain logistics process, the color of chilled chicken will change over time. In this study, a Konica Minolta portable spectrophotometer (CM-700d, Japan) was used to measure the color of chilled chicken on the surface of the chicken sample. Color is represented by CIE L* (luminosity value), a* (red value) and b* (yellow value). The calculation can be obtained by Equation (1):

(1) $$\Delta E=\sqrt{{\left(\Delta L^{*}\right)}^{2}+{\left(\Delta a^{*}\right)}^{2}+{\left(\Delta b^{*}\right)}^{2}}$$

Two random measurements were made directly on the surface of the sample and the average value was used for statistical analysis. The area selected for color measurement should not have obvious defects, as these defects may affect uniform color readings.

#### 2.3.3. Texture Profile Analysis

The TPA (Texture Profile Analysis) is an indicator of changes in the physical characteristics of chilled chicken, and their changes have a great correlation with quality [34]. The TA-XT PLUS Texture Analyzer (Stable Micro Systems, Surrey, UK) was used to test the hardness, stickiness, and chewiness. The sample was cut along the direction of the muscle fibers into a circle with a thickness of ~10 mm and a cross-sectional area of 10 cm^2^, while the sample weight was controlled at ~10 g and the compression of the sample was in the range of 20–90%. The probe used in the test was a P/5 flat-bottomed cylindrical probe with a pre-pressure of 5 g. The five-point sampling method was used to measure the TPA index and maintain a constant speed of 1.00 mm/s during the measurement. Each sample was tested three times, and the average value was taken as the various indicators.

#### 2.3.4. TVB-N

A volatile base nitrogen rapid detector (Foss Kjeltec^TM^ 8400, Beijing Hengrui Scientific Instrument Co., Ltd. Beijing, China) was used to determine the volatile base nitrogen value as the storage time changed. Thirty pieces of chilled chicken breasts were measured five times, as the initial TVB-N value of chilled chicken. The chicken was placed at different constant temperature incubators, the TVB-N content was detected according to different time intervals.

#### 2.3.5. Sensory Evaluation

Sensory evaluation and identification is a common and flexible method to identify the sensory traits (color, smell, and taste) of foods by people’s senses (eyes, nose, tongue, etc.) [35]. The evaluation team consisted of 10 food-related graduate students who were well trained in the field as evaluators. They evaluated and recorded the various indicators independently and could not communicate with each other. Sensory evaluation adopted a five-point system, and the total evaluation was divided into the average value of the sum of the other indicators. The specific evaluation standard is shown in Table 2.

#### 2.3.6. Statistical Analysis

The SPSS (International Business Machines Corporation, version 26.0, New York, NY, USA) was used to analyze all collected data by single-factor analysis of variance (ANOVA) at a significance level of 95% for quality analysis of chilled chicken at different temperatures. Besides, Duncan’s multiple comparison test was followed while significant differences were calculated at *p* ≤ 0.05 level for all analyses statistically.

## 3. Results and Discussion

### 3.1. Sensor Performance Evaluation

**Figure 3:**
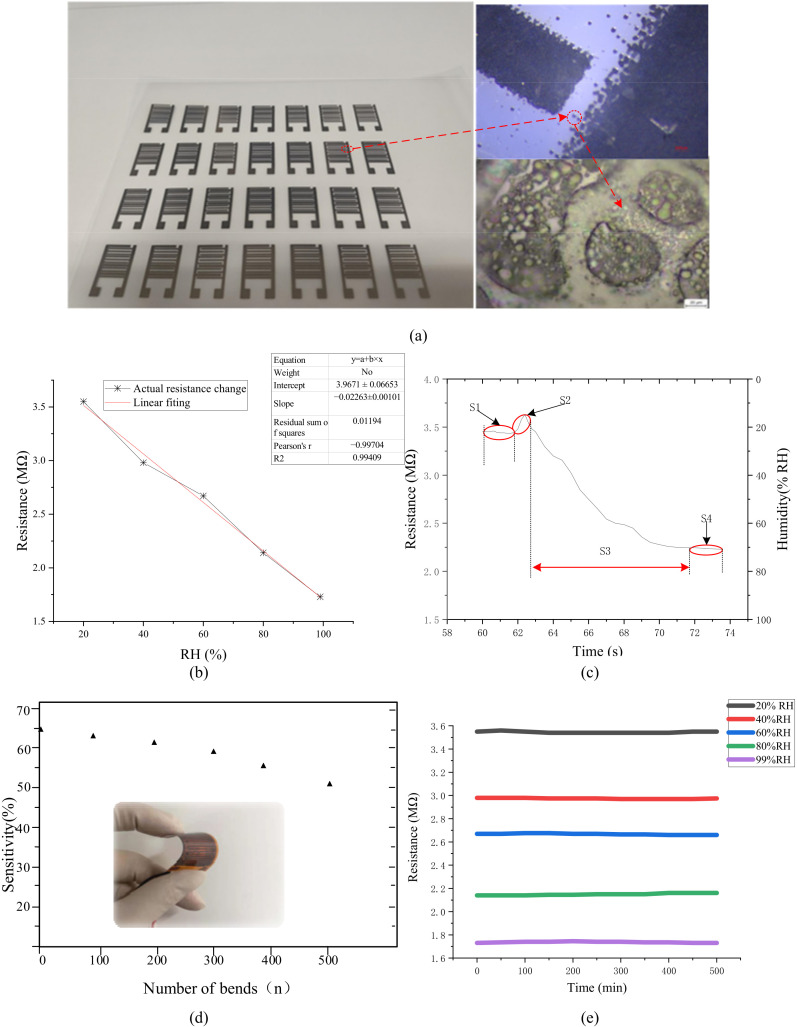
Sensor performance evaluation: (**a**) Sensor microstructure characterization; (**b**) Sensor static characteristic test; (**c**) Sensor dynamic response characteristic test; (**d**) Sensor flexibility test; (**e**) Sensor stability test.

The printed flexible humidity sensor array and its microstructure are shown in Figure 3a. The sensing layer material was uniformly attached to the interdigital electrode made of nano-silver, and its surface was distributed with many micro-pores with a diameter of about 1 μm. These pores increase the adsorption probability of water molecules on the surface of the sensing layer and improve the sensitivity of the flexible humidity sensor.

Static and dynamic calibration curves of the flexible humidity sensor are shown in Figure 3b,c, respectively. The resistance value of the sensor decreases with the increase of relative humidity, and the linear regression coefficient R^2^ of the fitting line reaches 0.99409, indicating that the resistance of the flexible humidity sensor has a good linear correspondence with relative humidity. The dynamic response process of the sensor can be roughly divided into four stages:

S1: The sensor had a stable output value in a low humidity environment;
S2: The airflow fluctuation in the container caused the sensor output to fluctuate;
S3: Due to the mixing of wet and dry air, the water molecules contacted by the sensor increase, and its output changed rapidly;
S4: Under stable humid air action, the sensor continuously responded and finally reached a stable output value.

Figure 3d,e, respectively, show the sensor flexibility test with different bending times and the stability test at different humidity. When the sensor was bent 90°, the sensor sensitivity response value dropped by 3.70%, 7.40%, 12.96%, 16.67% and 24.07% after different bending, compared with 54% of the sensor without bending. When the number gradually increased to 500 times, the response characteristics of the flexible sensor decreased significantly, and the flexibility of the sensor needs to be further improved. The fabricated flexible sensor showed good stability under five different relative humidity conditions. However, the stability of the flexible sensor at high relative humidity is better than that of low relative humidity, and it is still in the acceptable range on the whole.

Compared to the conventional sensor occupying the limited volume, the flexible humidity sensor with improved size and weight can be affixed in small packages to effectively increase the utilization of packages due to its good flexibility and ductility. Simultaneously, it can maintain excellent mechanical and sensing properties even under high-strength bending, stretching, and torsion, etc. Although the proposed flexible sensor has unique advantages in many fields, most of the current flexible sensors are still in the prototype development stage. The conventional humidity sensor still has better measuring accuracy and lower cost under the current situation. The multi-parameter monitoring equipment realized by integrating flexible sensors and rigid sensors in IoT can effectively deal with various abnormal situations in the cold chain process. To further optimize the performance of the monitoring device, it is considered to use high-performance, low-cost, lightweight flexible sensors such as flexible temperature and gas sensors. Integration with RFID technology is also considered to achieve efficient energy collection and low-power transmission in the future.

### 3.2. Critical Data Analysis and Evaluation

#### 3.2.1. Environment Parameter Data Analysis

**Figure 4:**
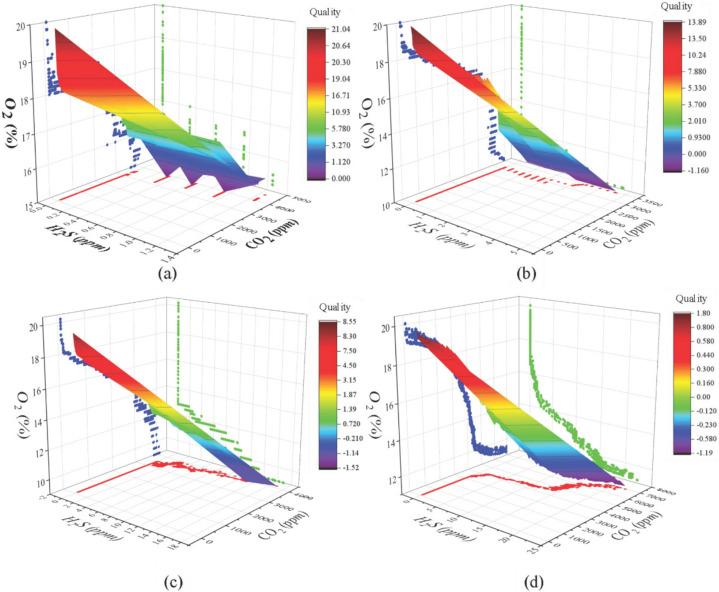
Quality change affected by gas concentration at different temperatures: (**a**) 0 °C; (**b**) 4 °C; (**c**) 8 °C; (**d**) 20 °C.

The quality change affected by gas concentration is shown in Figure 4 when chilled chicken samples were stored at different temperatures. The gas concentration changes have a higher similarity, and their simultaneous changes can indicate the chilled chicken quality. At different temperatures, the concentration of carbon dioxide maintains a slow increase in the initial stage and linearly changes in the corruption stage while the O_2_ content changes non-linearly; Meanwhile, H_2_S remained at 0 in the initial stage. When the signal was detected, the content of H_2_S gradually increased exponentially. With the increase of CO_2_ and H_2_S content and the decrease of O_2_, the chicken quality will gradually decrease with color from red to purple.

At 0 °C, 4 °C, and 8 °C, the oxygen content decreased rapidly in the first 3 h at the same time. Combined with humidity fluctuations, the reason is that the high humidity environment interferes with the accuracy of the oxygen sensor. Meanwhile, the metabolic rate of microorganisms decreases under low temperature, which leads to a decrease in the rate of oxygen decline and delays the deterioration of quality. The experimental results and correlation analysis (Table 3) show that, except that the correlation is not obvious at 0 °C, oxygen is significantly negatively correlated with chilled chicken quality, while carbon dioxide and hydrogen sulfide are significantly positively correlated. The main reason is that the chilled chicken has always been fresh at 0 °C, and the gas indicators have not changed obviously, especially the hydrogen sulfide has not been detected for a long period.

#### 3.2.2. Quality Indicator Data Analysis

**Figure 5:**
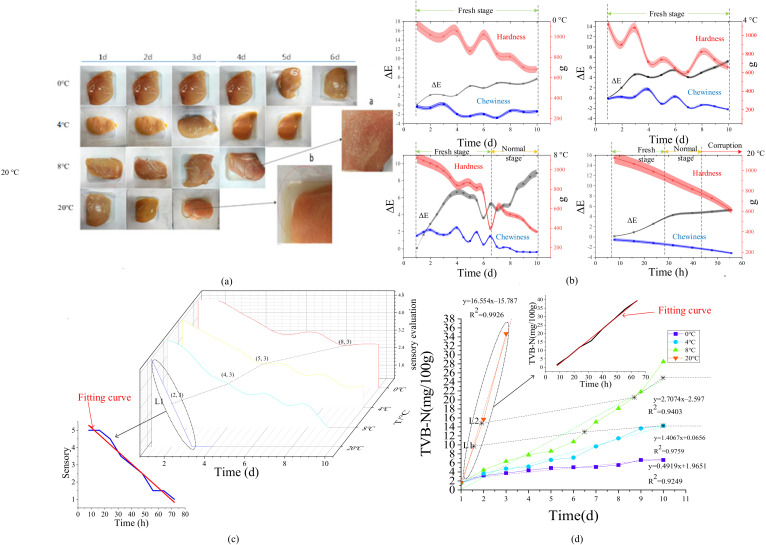
Different quality characteristics of chilled chicken. (**a**) Color Change; (**b**) TPA (color, hardness, and chewiness) changes at different temperatures; (**c**) Sensory evaluation; (**d**) TVB-N.

As shown in Figure 5a, it is seen that the quality of chickens refrigerated at a low temperature has almost no change in the first 4–5 days, which is difficult to distinguish by color difference. For chicken stored at higher temperatures, especially at 20 °C, the change was obvious. The color became dark, a lot of juice was exuded, pungent odor and sticky substance appeared, and the tissue became soft and had no elasticity. The texture profile measured on chicken samples stored at different temperatures is shown in Figure 5b. The luminosity value L*, red value a*, and yellow value b* of chilled chickens decreased with the extension of storage time, that is, the color became darker and blacker. Among them, at the temperature of 8 °C, the value ΔE increased immediately in the first four days, but it dropped from the fourth to the seventh day. The color became brighter, which is consistent with the appearance of a sticky white substance on the eighth day in the color changes. In summary, the color difference value ΔE changes linearly and has a negative correlation with the quality change of chilled chicken. The changes in hardness and chewiness of chilled chicken at different temperatures are also shown in Figure 5b, respectively. Both of them show an obvious positive correlation with the quality of chilled chicken, which can be used as one of the quality indicators.

Therefore, considering the color difference, hardness, and other indicators comprehensively, the result of sensory evaluation is shown in Figure 5c. It can be seen that three points are an obvious cut-off point. When the score is higher than three points, the chilled chicken is fresher, shiny, and has no peculiar smell. With the extension of storage time, the chilled chicken became dull, the normal smell disappeared, and a peculiar smell appeared, the chicken tissue was loose, inelastic, and enter the stage of corruption. To analyze the chicken quality comprehensively, it is necessary to further combine other indicators for in-depth analysis. The TVB-N content change under different temperature conditions is shown in Figure 5d. According to the fitting effect, the linear correlation results of TVB-N at 20 °C, 8 °C, 4 °C, and 0 °C are 0.9926, 0.9403, 0.9759, 0.9249, respectively, which proves that the fitting effect has an obvious quality indication.

#### 3.2.3. Critical Data Correlation Analysis

The correlation results of each parameter under different temperature conditions are shown in Table 3, H_2_S and CO_2_ have a high positive correlation with TVB-N and a high negative correlation with sensory evaluation. Meanwhile, O_2_ and humidity have a high positive correlation with sensory evaluation and a certain negative correlation with TVB-N. On the other hand, the hardness, chewiness and color difference are not significantly correlated with environmental parameters under low-temperature conditions. Therefore, H_2_S, CO_2_, O_2_, and humidity are selected as the input of the neural network, and TVB-N and sensory evaluation indicators are used as the output for the characterization of chicken quality.

### 3.3. Quality Evaluation Standard

**Figure 6:**
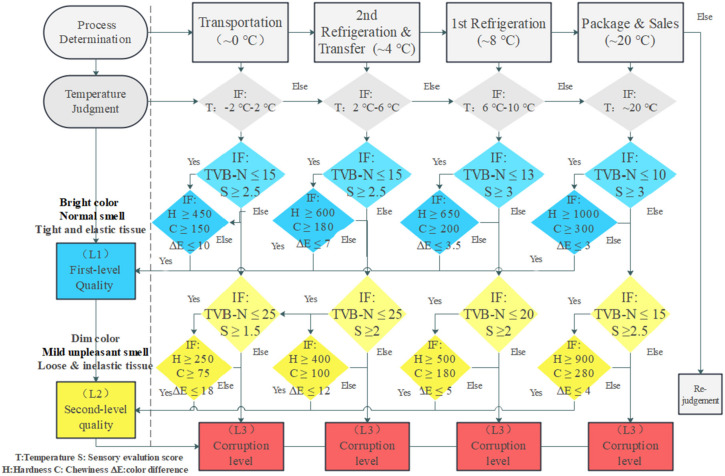
The quality evaluation standard based on knowledge rules under different conditions.

The quality evaluation standard of chilled chicken based on knowledge rules under different conditions is shown in Figure 6. Since temperature fluctuation is the most important factor affecting chilled chicken quality change, while other environmental indicators are more used to predict chicken quality, they were not included in the construction of quality evaluation standards. According to the established knowledge rules database, the quality evaluation level of chilled chicken is roughly divided into three stages, namely L1, L2, and L3, which correspond to the first-level quality, second-level quality and the corruption level, respectively. Based on the knowledge rules and the statistical analysis results obtained from the collected data, the cold chain process and the corresponding temperature requirements were determined, and all quality indicators were classified and judged according to the importance of the correlation results for the final quality evaluation standard. As shown in Table 3, TVB-N and sensory evaluation have a significant correlation with the final quality and shelf life, so they occupy higher weight in the quality evaluation standard. Simultaneously, other indicators (such as hardness, color, etc.) are also indispensable, which are used for secondary quality evaluation to make the evaluation standard more comprehensive and detailed. According to the extracted knowledge rules, the quality evaluation standard is established to effectively evaluate the quality and feedback the evaluation results to the knowledge rule database to provide relevant diagnostic information and knowledge rules for subsequent dynamic prediction. The specific knowledge rules are as follows:

**Rule** **1:** The cold chain process is determined, and the corresponding requirements are selected.
**Rule** **2:** The first stage of quality level judgment. According to the selected condition, judge whether the TVB-N content and sensory evaluation score meet the requirements. Otherwise, the quality level judgment of the next stage will be carried out.
**Rule** **3:** If the requirements are met, judge whether the color difference, hardness, and chewiness meet the requirements. Otherwise, the quality level judgment of the next stage will be carried out.
**Rule** **4:** If all the requirements are met, the chilled chicken is considered as first-level quality with bright color, tight and elastic tissue, and a normal smell.
**Rule** **5:** The second stage of quality level judgment. Repeat the judgment method of Rule 2–4. When TVB-N, sensory score, color difference, and all other indicators meet the corresponding requirements, chilled chicken is considered in second-level quality with dim color, loose and inelastic tissue, and mild unpleasant smell.
**Rule** **6:** If all the requirements are not met, it is considered that the chilled chicken has entered the corruption level with terrible quality.

In different conditions, the time to reach three levels is also different. The quality change of chilled chicken is most obvious at 20 °C while the quality validity period of chilled chicken during package and sales is very short (less than two days). Compared with other cold process conditions, the quality classification requires stricter TVB-N content and sensory evaluation scores, since the chilled chicken at 20 °C did not produce much TVB-N in a short time and the sensory evaluation was not obvious. In contrast, the sensory evaluation scores of chilled chickens at 0 °C are influenced by environmental factors such as air-drying due to longer shelf life, so the sensory evaluation scores are lower, and the content of TVB-N is relatively higher. Therefore, it is better to ensure that the cold chain temperature of chilled chicken is maintained at 4 °C to maximize the quality. Besides, the maximum temperature should not exceed 8 °C (the quality validity period is higher than 8 days) while the minimum temperature should not be lower than 0 °C (to ensure its good texture and flavor).

### 3.4. Evaluation of Quality Prediction Model

**Figure 7:**
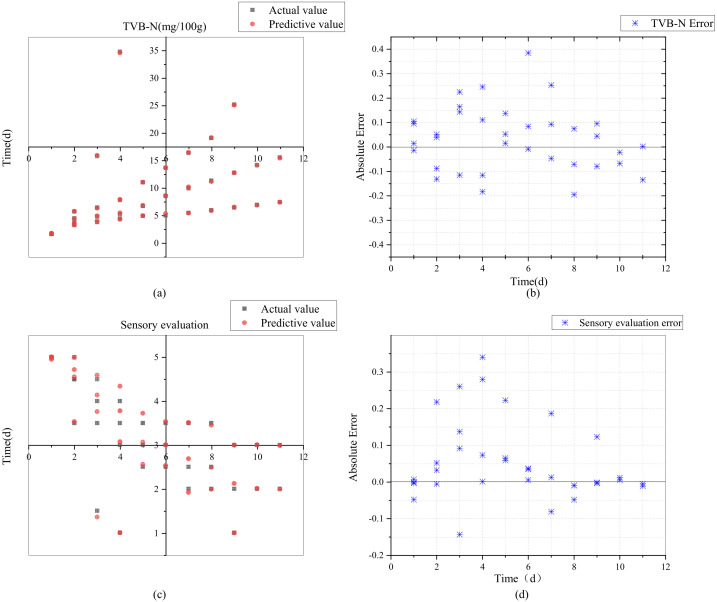
The performance analysis of the improved prediction model. (**a**) The absolute error of TVB-N; (**b**) The relative error of the TVB-N; (**c**) The absolute error of sensory evaluation; (**d**) The relative error of sensory evaluation.

To demonstrate the fitting and prediction ability of the model, according to the construction step of the prediction model in Section 2.3, the concentration of humidity, O_2_, CO_2_, and H_2_S were used as the input of the prediction model. Meanwhile, the TVB-N and sensory evaluation indicators were respectively used as the output of the prediction model. Two output indicators were comprehensively considered to obtain the chilled chicken quality evaluation and prediction. The model was trained and tested for a total of 9000 sets of data at four different temperatures: 70% of the data was used as the training set, 15% of the data was used as the test set and 15% of the data was used as the validation set of the model. The performance analysis of the improved prediction model based on the knowledge rules between the predicted value and the true value is shown in Figure 7. The absolute error of the two indicators changed in the interval [−0.1948, 0.3846] and [−0.183, 0.3405], respectively. The changing trend of the two indicators presents a strong consistency of the predictive value estimation. Meanwhile, the relative error of the TVB-N and sensory evaluation prediction models are less than 9.5% and 8.5%, respectively, which demonstrated that the model had higher prediction accuracy and lower error. Additionally, the correlation coefficient R between the predicted value and the actual value of the model are all higher than 0.99, which indicated that the quality coupling model based on the multi-parameter combination had high prediction accuracy in three stages. In summary, by analyzing the prediction results of the model, the complementation and combination of multiple parameters can greatly improve the prediction results of chilled chicken quality, which can effectively reflect changes in shelf life.

### 3.5. Experimental Verification and Model Comparison Evaluation

**Figure 8:**
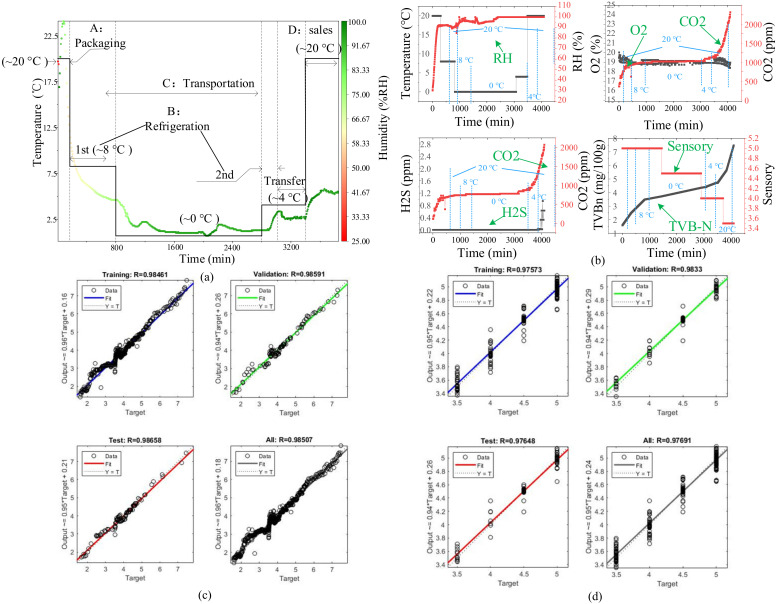
The actual cold chain of chilled chicken and simulation verification. (**a**) Actual cold chain; (**b**) Critical parameters and indicators change; (**c**) TVB-N prediction verification; (**d**) Sensory evaluation prediction verification.

The actual cold chain (East China Meat Market, Jiangsu-Beijing) real-time tracking monitoring results are shown in Figure 8a. The whole process took about 3000 min from packaging to refrigeration in a laboratory incubator. The temperature fluctuated within the range of 20–30 °C during packaging and dropped sharply from 25 °C to 8 °C during the 1st refrigeration. The temperature in the cold chain transportation stage was maintained at about 0 °C and was constant at about 3 °C during the 2nd refrigeration. Among them, a series of uncertain factors in the logistics process, such as refrigeration equipment failure, transportation instability, etc., would cause temperature fluctuations. Meanwhile, the humidity was always in a high humidity state above 90% RH since it rose from 50% RH.

Based on the temperature changes in the actual cold chain process, variable temperature experiments were designed to verify the quality evaluation and prediction models based on knowledge rules. The changes in environmental parameters and quality indicators are shown in Figure 8b. Various parameters and indicators are highly similar to their changing trends at different temperatures. Humidity, O_2_, CO_2_, and H_2_S were used as inputs to predict chicken quality, and the final result (Figure 8c,d) shows that the correlation coefficient R is greater than 0.9785, confirming the excellent performance of the model.

Table 4 provides a comprehensive comparison of the traditional model and optimized model in detail. Compared with the traditional model, the proposed prediction model integrates flexible humidity sensors into intelligent IoT monitoring, which achieves shorter response and recovery time and higher accuracy while realizing multi-parameter monitoring. Simultaneously, the traditional evaluation method only considers the influence of TVB-N content and sensory evaluation on quality in any case. The improved model performs data statistical analysis on collected relevant data and determines the cold chain process and requirements under different conditions. The quality evaluation based on knowledge rules is established to meticulously evaluate the chilled chicken indicators under different conditions and provide more accurate information and knowledge rules for the prediction model. After error analysis and calibration verification, it is found that the critical parameter-quality prediction model can accurately predict chicken quality, which verifies that the optimization of the model has reached the expected demand.

Participants’ and other cold chain managers’ suggestions are also shown in Table 4. The optimized monitoring equipment still has the problem of weak signal transmission and high battery power consumption, resulting in poor sustainability of equipment applications. It is also suggested that the future equipment will integrate more flexible micro-sensors, which can be pasted on the express package to reduce sensor size and the economic cost of the equipment. Other critical parameters (such as ammonia) and quality indicators (such as pH) can also be considered to improve the credibility of quality evaluation and the accuracy of quality prediction.

## 4. Conclusions

This paper integrated multi-sensor non-destructive monitoring methods, the quality evaluation level and intelligent dynamic prediction model based on knowledge rules. The performance optimization and demonstration of the intelligent dynamic quality prediction for chilled chicken cold chain had made important contributions to wider applications of IoT in food supply chain operation and management. The approaches and processes developed for the proposed model can be adopted by other researchers and practitioners in cold chain management. The following are the main conclusions of this research:

(1) A flexible humidity sensor was designed and fabricated through inkjet printing. The integrated flexible and rigid sensors in IoT could effectively improve packaging effect and monitoring accuracy;
(2) Through the analysis of the critical points of the cold chain, micro-environmental factors and quality indicators, the relevant rules were extracted, and the collected data were statistically analyzed. Significant relevant indicators were constructed to build a knowledge rules database for subsequent quality evaluation standard and prediction;
(3) The intelligent dynamic quality prediction model based on knowledge rules and quality evaluation standards comprehensively evaluated the chicken quality in level and provided a high-precision prediction of quality.

## Figures and Tables

**Table 1:** Parameter performance of various sensors in chilled chicken transportation.

ID	Types	Measured Parameters	Measuring Range	Resolution	Accuracy	Power Consumption
1	AJD-O_2_	O_2_	0–25%	–	±0.5%	200 mW
2	AJD-CO_2_	CO_2_	0–5000 ppm	–	±50 ppm	25 mW
3	AJD-H_2_S	H_2_S	0–1000 ppm		±10 ppm	220 mW
4	ST11	Temperature	−40~80 °C	0.1 °C	±0.2 °C	1–30 uW
5	TH-2303	Humidity	0–99% RH	0.1% RH	±0.5% RH	1–30 uW

**Table 2:** The specific evaluation standard of chilled chicken.

Evaluation Standard	Color	Smell	Tissue
5	Bright red and shiny	Originally normal smell	Very tight and elastic
4	Light red and shiny	Normal smell	Tight and elastic
3	Light red and dull	Normal smell becomes lighter	Loose and inelastic
2	Dim color	Normal smell disappeared and slightly peculiar	More loose
1	Dark brown with some green	Smelly or ammonia smell	Very loose

**Table 3:** Correlation matrix analysis of different indicators.

		RH	H_2_S	O_2_	CO_2_	TVB−N	Sensory	ΔE	Hardness	Chewiness
20 °C	RH	1								
	H_2_S	0.489	1							
	O_2_	−0.588	−0.942 *	1						
	CO_2_	0.639	0.971 *	−0.987 *	1					
	TVB−N	0.779	0.994 **	−0.941 *	0.980 *	1				
	Sensory	−0.812 *	−0.852 *	0.948 *	−0.952 *	−0.886 *	1			
	ΔE	0.937 *	0.771	−0.799	0.890	0.918	−0.969	1		
	Hardness	−0.821 *	−0.905 *	0.923 *	−0.975 ^*^	−0.988 *	1.000 **	−0.969 *	1	
	Chewiness	−0.811	−0.912 *	0.930 *	−0.979 *	−0.990 *	1.000 *	−0.964	1.000 *	1
8 °C	RH	1								
	H_2_S	0.153	1							
	O_2_	−0.571	−0.834 *	1						
	CO_2_	0.481	0.888 *	−0.956 **	1					
	TVB−N	0.843 *	0.970 *	−0.933 **	0.978 **	1				
	Sensory	−0.868 *	−0.858 *	0.921 **	−0.929 **	−0.949 **	1			
	ΔE	0.548	0.654	−0.662	0.525	0.529	−0.733 *	1		
	Hardness	−0.565	−0.532	0.949 **	−0.981 **	−0.981 **	0.952 **	−0.743 *	1	
	Chewiness	−0.251	−0.521	0.817 **	−0.905 **	−0.877 **	0.787 *	−0.486	0.857 **	1
4 °C	RH	1								
	H_2_S	0.153	1							
	O_2_	−0.571	−0.786 *	1						
	CO_2_	0.481	0.824	−0.940 **	1					
	TVB−N	0.723	0.911 *	−0.978 **	0.981 **	1				
	Sensory	−0.686	−0.802	0.950 **	−0.890 **	−0.955 **	1			
	ΔE	0.505	0.540	−0.877 **	0.864 **	0.875 **	−0.841 **	1		
	Hardness	−0.532	−0.325	0.749 *	−0.592	−0.692 *	0.790 **	−0.572	1	
	Chewiness	−0.268	−0.367	0.652 *	−0.776 **	−0.743 *	0.676 *	−0.520	0.346	1
0 °C	RH	1								
	H_2_S	0.226	1							
	O_2_	−0.963 **	−0.522	1						
	CO_2_	0.320	0.763 *	−0.357	1					
	TVB−N	0.878 *	0.782	−0.723 *	0.840 **	1				
	Sensory	0.841 *	−0.685	0.615 *	−0.759 **	−0.945 **	1			
	ΔE	0.494	0.398	−0.600	0.822 **	0.924 **	−0.893 **	1		
	Hardness	−0.505	−0.288	0.553	−0.859 **	−0.897 **	0.834 **	−0.936 **	1	
	Chewiness	−0.366	−0.285	0.511	−0.175	−0.532	0.657 *	−0.521	0.362	1

* indicates that the linear correlation is significant, ** indicates that the linear correlation is more significant. *p* ≤ 0.05.

**Table 4:** Comprehensive comparison of the traditional model and proposed model.

Model Performance	Sensors Performance and Environmental Parameters Evaluation						Quality Analysis and Evaluation								Prediction Model Evaluation
	Monitoring Parameters	Temperature	Humidity	CO_2_	O_2_	H_2_S	(L1) First-Level Quality				(L2) Second-Level Quality				
							0 °C	4 °C	8 °C	20 °C	0 °C	4 °C	8 °C	20 °C	
Previous monitoring method	Temperature and Humidity	Range: −40–120 °C Accuracy: ±0.4 °C	Range: 0-100% RH Accuracy: ±3% RH	None	None	None	None				None				None
Improved model	Temperature humidity CO_2_ O_2_ H_2_S	Range: −40–80 °C Accuracy: ±0.3 °C	Range: 0–100% RH Accuracy: ±1% RH	Range: 0–50% vol accuracy: ±2% vol Response time <25 s	Range: 0–30% vol accuracy: ±1% vol Response time <25 s	Range: 0–100 ppm accuracy: ±1 ppm Response time <25 s	TVB-N: ≤15	TVB-N: ≤15	TVB-N: ≤13	TVB-N: ≤10	TVB-N: ≤25	TVB-N: ≤25	TVB-N: ≤20	TVB-N: ≤15	Relative error < 8% R^2^ > 0.996
							S: ≥ 2.5	S: ≥ 2.5	S: ≥ 3	S: ≥ 3	S: ≥ 1.5	S: ≥ 2	S: ≥ 2	S: ≥ 2.5	
							H ≥ 450	H ≥ 600	H ≥ 650	H ≥ 1000	H ≥ 250	H ≥ 400	H ≥ 500	H ≥ 900	
							C ≥ 150	C ≥ 180	C ≥ 200	C ≥ 300	C ≥ 75	C ≥ 100	C ≥ 180	C ≥ 280	
							ΔE ≤ 10	ΔE ≤ 7	ΔE ≤ 3.5	ΔE ≤ 3	ΔE ≤ 18	ΔE ≤ 12	ΔE ≤ 5	ΔE ≤ 4	
Advantages	Multiple critical parameters monitoring	Better accuracy and traceability Real-time, non-destructive, and online monitoring Integrating flexible sensor with better performance					Different quality evaluation standard under different temperature Detailed and comprehensive quality evaluation								Predict effectively and accurately without contact and contamination
Suggestions	More critical parameters	Develop flexible, passive and small-size sensors for smaller packages					The scoring criteria of sensory evaluation still need to be refined and improved								Accuracy can still be improved
